# Tianhuang formula reduces the oxidative stress response of NAFLD by regulating the gut microbiome in mice

**DOI:** 10.3389/fmicb.2022.984019

**Published:** 2022-09-21

**Authors:** Duosheng Luo, Ling Yang, Huiting Pang, Yating Zhao, Kunping Li, Xianglu Rong, Jiao Guo

**Affiliations:** ^1^Guangdong Metabolic Diseases Research Center of Integrated Chinese and Western Medicine, Key Laboratory of Glucolipid Metabolic Disorder, Ministry of Education of China, Guangzhou, China; ^2^Institute of Chinese Medicine, Guangdong Pharmaceutical University, Guangdong TCM Key Laboratory for Metabolic Diseases, Guangzhou, China

**Keywords:** NAFLD, gut microbiome, oxidative stress, Tianhuang formula, fecal microbial transplantation

## Abstract

**Background:**

The gut microbiome affects the occurrence and development of NAFLD, but its mechanism has not yet been fully elucidated. Chinese medicine is a new treatment strategy to improve NAFLD by regulating the gut microbiome. Tianhuang formula (TH) has been proved to have a lipid-lowering effect in which constituents of ginsenoside Rb1, ginsenoside Rg1, ginsenoside Rb, ginsenoside Re, and ginsenoside R1 from *Panax notoginseng* and berberine, palmatine, and coptisine from *Coptis chinensis* have low drug permeability, which results in poor intestinal absorption into the human body, and are thus able to come into contact with the gut microflora for a longer time. Therefore, it might be able to influence the gut microbial ecosystem, but it still needs to be investigated.

**Method:**

The characteristics of the gut microbiome were represented by 16S rRNA sequencing, and the metabolites in intestinal contents and liver were discovered by non-targeted metabolomics. Correlation analysis and fermentation experiments revealed the relationship between the gut microbiome and metabolites. Blood biochemical indicators, liver function indicators, and oxidation-related indicators were assayed. H&E staining and Oil Red O staining were used to analyze the characteristics of hepatic steatosis. RT-qPCR and western blotting were used to detect the expression of genes and proteins in liver tissues, and fecal microbial transplantation (FMT) was performed to verify the role of the gut microbiome.

**Results:**

Gut microbiome especially *Lactobacillus* reduced, metabolites such as 5-Methoxyindoleacetate (5-MIAA) significantly reduced in the liver and intestinal contents, the level of hepatic GSH and SOD reduced, MDA increased, and the protein expression of Nrf2 also reduced in NAFLD mice induced by high-fat diet (HFD). The normal diet mice transplanted with NAFLD mice feces showed oxidative liver injury, indicating that the NAFLD was closely related to the gut microbiome. TH and TH-treated mice feces both can reshape the gut microbiome, increase the abundance of *Lactobacillus* and the content of 5-MIAA in intestinal contents and liver, and improve oxidative liver injury. This indicated that the effect of TH improving NAFLD was related to the gut microbiome, especially *Lactobacillus*. 5-MIAA, produced by *Lactobacillus*, was proved with fermentation experiments *in vitro*. Further experiments proved that 5-MIAA activated the Nrf2 pathway to improve oxidative stress in NAFLD mice induced by HFD. TH reshaped the gut microbiome, increased the abundance of *Lactobacillus* and its metabolite 5-MIAA to alleviate oxidative stress, and improved NAFLD.

**Conclusion:**

The study has demonstrated a mechanism by which the gut microbiome modulated oxidative stress in NAFLD mice induced by HFD. The traditional Chinese medicine TH improved NAFLD by regulating the gut microbiome, and its mechanism was related to the “*Lactobacillus*-5-MIAA-Nrf2” pathway. It provided a promising way for the intervention of NAFLD.

## Introduction

Non-alcoholic fatty liver disease (NAFLD) is a liver manifestation of metabolic syndrome with a multi-factor clinicopathological state (Zhang et al., 2021). Lipids accumulate in cells and then form lipid droplets (steatosis) in the cytoplasm of hepatic cells. NAFLD has become a serious public health problem (Sahini and Borlak, 2014). Regarding the pathogenesis of NAFLD, insulin resistance, and the “Two-Hit” theory, oxidative stress, apoptosis, inflammation, and fibrosis caused by metabolic disorders have been paid more attention in the past (Patil et al., 2020). In recent years, emerging metagenomics has confirmed that the gut microbiome is highly related to lipid metabolism disorders and is an independent pathogenic factor. Regulating the gut microbiome to improve NAFLD has attracted the attention of researchers. Oxidative stress is closely related to the pathogenesis of NAFLD (Begriche et al., 2013). Nuclear factor-erythroid 2-related factor 2 (Nrf2), as a classic antioxidant pathway in the liver, activates the oxidative stress defense system by inducing antioxidants and detoxification enzymes and protects cells from various oxidative injuries (Itoh et al., 1997). The downstream of the Nrf2 pathway, NQO1, is a cytoprotective factor that is widely expressed and fights oxidative stress by removing superoxides, retaining various endogenous antioxidants and catalyzing reduction metabolism (Ross, 2004). In addition, the intracellular glutathione (GSH) and heme oxygenase 1 (HO-1) are all activated by Nrf2 to resist oxidative stress (Gong et al., 2002). Many studies have shown that the Nrf2 pathway plays an indispensable role in the oxidative stress response during the progression of NAFLD (Lee et al., 2014). Studies have proved that oxidative stress is more serious which leads to the evolution of NAFLD to NASH in the Nrf2 knockout mice (Chowdhry et al., 2010).

The gut microbiome is an indispensable “organ” in the human intestine and is called the “second genome” of humans (Qin et al., 2010; Salazar et al., 2017). More and more studies have shown that the gut microbiome is a “microbial organ” in the body, through its components, metabolites, and derivatives (Dalile et al., 2019), as well as the translocation of pathogenic commensal bacteria (Meisel et al., 2018), participate in the regulation of host metabolism (Tilg et al., 2020), immunity (Belkaid and Harrison, 2017), endocrine (Rastelli et al., 2019), nerve (Sharon et al., 2016), and systemic physiological processes, thereby affecting obesity (Le Chatelier et al., 2013), diabetes (Qin et al., 2010), cardiovascular disease (Schiattarella et al., 2017), autoimmune and inflammatory diseases (Clemente et al., 2018), mental and neurological diseases (Cryan et al., 2020), and other diseases. The relationship between NAFLD and the gut microbiome has also become the focus of researchers. A recent study by Xie et al. found that vine tea polyphenols prevented western diet-induced NAFLD by regulating fatty acid oxidation and adipogenesis, hepatic oxidative stress, and gut microbiome, confirming the possibility of improving oxidative stress by regulating the gut microbiome in NAFLD (Xie et al., 2020). Many studies have suggested a role for the gut microbiome in modulating NAFLD; however, the mechanisms by which they impact NAFLD remain largely unknown.

Some active ingredients from traditional Chinese medicine have been shown to improve NAFLD by regulating inflammation, lipid production, insulin sensitivity, mitochondrial dysfunction, autophagy, and gut microbiome (Zhou et al., 2021). For example, the polysaccharide extracted from broken Ganoderma lucidum spore powder alleviates obesity, hyperlipidemia, and inflammation in HFD-induced mice via regulating the gut microbiome (Sang et al., 2021). The composition of geniposide and chlorogenic acid improves NAFLD induced by HFD (Chen et al., 2021). Ginkgo extract GBE50 improves HFD-induced lipid accumulation and hepatic steatosis in NAFLD mice, and its mechanism may be related to activating IRS-1 and inhibiting NF-κB and ERS (Li L. et al., 2021). TH is composed of *Panax notoginseng* and *Coptis chinensis* which is a patented Chinese medicinal prescription with hypolipidemic effects, based on the theory of traditional Chinese medicine “TiaoGan QiShu HuaZuo” (Specialty Committee of Metabolic Diseases of WFCMS, 2019). Studies have shown that TH has the effect of lowering lipid levels (Luo et al., 2016, 2019; Li K. P. et al., 2021). However, constituents of TH were ginsenoside Rb1, ginsenoside Rg1, ginsenoside Rb, ginsenoside Re, and ginsenoside R1 from *P. notoginseng* and berberine, palmatine, and coptisine from *C. chinensis* whose concentrations are very low in the serum taking orally. Therefore, it remains unclear whether gut microbiota plays an important role in regulating lipid metabolism disorders by TH.

In this study, we found that TH and mice feces of treatment with TH both modulated the gut microbiome, increased the abundance of *Lactobacillus* and the content of 5-MIAA in intestinal contents and liver, and improved oxidative liver injury. This indicated that the effect of TH improving NAFLD was related to the gut microbiome especially *Lactobacillus* and its metabolite 5-MIAA. And further, 5-MIAA was identified as a novel *lactobacillus*-derived small molecule capable of activating hepatic Nrf2. TH modulated the gut microbiome, increased the abundance of *Lactobacillus* and its metabolite 5-MIAA to alleviate oxidative stress, and improve NAFLD. These data addressed key gaps in our understanding of the gut-liver axis and provided further insight into the role of the gut microbiome in hepatic health and homeostasis.

## Materials and methods

### Preparation and chemical constituents of Tianhuang formula

Herbs in TH [*P. notoginseng* and *C. chinensis]* were provided by Zhixin Chinese Herbal Medicine Co., Ltd. (Guangzhou, China). TH was obtained from the Institute of Guangdong Metabolic Diseases Research Center of Integrated Chinese and Western Medicine, Guangdong Pharmaceutical University. TH was prepared and quantitated as previously described (Li K. P. et al., 2021). In brief, powdered *P. notoginseng* (400 g) and *C. chinensis* (400 g) were separately extracted triply with 70% ethanol at 80°C under reflux, each time for 2 h. The extract solution was concentrated in a rotary evaporator to remove ethanol, and then dissolved in water and purified using D101 macro-porous resin (Lanxiao, Xi’an). The resulting purified extract was dried in a vacuum at 60°C. The quantitative profiling of TH was performed on a U3000 HPLC with a DAD detector (Dionex, United States). The chromatography separation was carried out using a Kromasil C18 column (4.5 × 250 mm, 5 μm in particle size) according to the Pharmacopoeia of the People’s Republic of China (Ch. P. 2020), and the data were recorded and analyzed on Chromeleon Console workstation. Finally, the contents of eight active components in TH, namely, ginsenoside Rg1, ginsenoside Rb1, ginsenoside Rd, ginsenoside Re, notoginsenoside R1, berberine, coptisine, and palmatine, were quantified.

### Animals and experimental design

All the animal experiments were approved by the Animal Ethical Committee of Guangdong Pharmaceutical University (GDPULACSPF2017079). Specific pathogen-free (SPF) male C57BL/6J Narl mice, 3 to 4 weeks of age, were purchased from the Guangdong Medical Laboratory Animal Center. All mice were housed in a temperature-controlled room at 24°C ± 2°C, with a humidity of 60 to 70%, and 12 h of light and darkness alternated.

All mice were randomly assigned to control group (CON) and test group. The test group was fed a high-fat diet (60 fat, 20 protein, 20% carbohydrate; Research Diets, D12492) for 14 weeks, then the test group mice were divided into three groups, including the model group (vehicle), TH-treated group (100 mg/kg/day, TH), and atorvastatin group(1.5 mg/kg/day, AT) for 6 weeks. Before mice were sacrificed, feces were collected in an SPF feeding environment one day. Overnight fasted mice were sacrificed after anesthesia with 1% pentobarbital sodium solution by intraperitoneal injection. Samples were collected and immediately frozen at −80°C for further analysis.

### Biochemical analysis

Serum was collected for detection of the total cholesterol (TC), triglycerides (TG), low-density lipoprotein cholesterol (LDL-C), alanine transferase (ALT), and aspartate transferase (AST). Malondialdehyde (MDA), glutathione (GSH), and superoxide dismutase (SOD) in the liver were analyzed using kits (Nanjing Jiancheng Technology Co., Ltd.), according to the manufacturer’s protocols.

### Histopathological observation of liver

Liver sections were fixed in 10% formalin overnight, embedded in paraffin, sectioned, and then stained with hematoxylin and eosin (H&E) for histological examination. For the detection of lipid accumulation, frozen liver sections were stained with oil red O according to a standard protocol (Nanjing Jiancheng Bioengineering Institute, Nanjing, China). Finally, liver sections were imaged at 200x magnification (Olympus, Tokyo, Japan). The data shown were from one representative experiment of three independent repeats.

### RNA extraction and quantitative real-time PCR analysis

Total RNA was isolated by using RNAiso Plus Reagent (Takara, Japan), and the concentration was measured based on the absorbance ratio of 260 nm/280 nm. Subsequently, 1 μg of total RNA was reverse-transcribed into complementary DNA with a first-strand synthesis kit (Takara, Japan). For each sample, PCR was performed in triplicate with SYBR Green reagents (Takara, Japan) and the LC480 platform (Roche, United States). The expression levels of the target genes were normalized to that of the internal control (GAPDH). The primer sequences were listed in Supplementary Table 1.

### Protein extraction and western blotting analysis

Proteins were extracted with radioimmunoprecipitation assay (RIPA) lysis buffer and centrifuged at 12,000 rpm and 4°C for 10 min. The protein lysates were separated by SDS-PAGE, subsequently transferred to PVDF membranes, and blocked for 2 h at room temperature. The polyvinylidene difluoride membranes were incubated with the specific primary antibodies of interest listed in Supplementary Table 2 and then with the appropriate secondary antibodies (1:1,000) for 2 h at room temperature. The blot was visually detected with an enhanced chemiluminescence substrate (Meilunbio, China) and a Bio-Rad GelDoc imaging system. The immunoblot analysis was performed using the Image J software.

### 16S rRNA sequencing of the gut microbiome

Fecal bacterial DNA was extracted using the E.Z.N.A.^®^ soil DNA Kit (Omega Bio-Tek, Norcross, GA, United States) according to the manufacturer’s protocols. The final DNA concentration and purification were determined by NanoDrop 2000 UV-vis spectrophotometer (Thermo Scientific, Wilmington, United States), and DNA quality was checked by 1% agarose gel electrophoresis. The V3-V4 hypervariable regions of the bacteria 16S rRNA gene were amplified with primers 338F (5′-ACTCCTACGGGAGGCAGCAG-3′) and 806R(5′-GGACTACHVGGGTWTCTAAT-3′) by thermocycler PCR system (GeneAmp 9700, ABI, United States). The PCR reactions were conducted using the following program: 3 min of denaturation at 95°C, 27 cycles of 30 s at 95°C, 30 s for annealing at 55°C, and 45 s for elongation at 72°C, and a final extension at 72°C for 10 min. PCR reactions were performed in a triplicate 20 μL mixture containing 4 μL of 5 × FastPfu Buffer, 2 μL of 2.5 mM dNTPs, 0.8 μL of each primer (5 μM), 0.4 μL of FastPfu Polymerase, and 10 ng of template DNA. The resulted PCR products were extracted from a 2% agarose gel and further purified using the AxyPrep DNA Gel Extraction Kit (Axygen Biosciences, Union City, CA, United States) and quantified using QuantiFluor™-ST (Promega, United States) according to the manufacturer’s protocol.

### Metabolomics analysis

About 100 mg (± 2%) of intestinal contents samples or 100 mg (± 2%) of liver samples added 2-chlorophenylalanine 80% methanol, then were shaken at 2,500 rpm for 10 min, and refrigerated at −20°C for 10 min. Then, the samples were centrifuged at 12,000 rpm for 10 min. Subsequently, the supernatant was reconstituted with 100 μL 50% methanol-water resolution and was transferred into a vial subjected for UPLC-MS/MS analysis. Take 20 μL of each sample to be tested and mix it into quality control (QC) sample for error correction. An ACQUITY UPLC HSS T3 column (1.8 μm 2.1 × 100 mm; Waters, United States) was used at 40°C column temperature. The injection volume was 2 μL in both positive and negative ion modes. The mobile phase consisted of 0.1% formic acid (Merck, Germany) in water as solvent A and 0.1% formic acid in acetonitrile (Merck, Germany) as solvent B. The flow rate of the mobile phase was 0.25 mL⋅min^–1^.

The mass spectrometric parameters were as follows: Electrospray ionized-ion source (ESI), positive ion spray voltage was 3.50 kV, negative ion spray voltage was 2.50 kV, sheath gas 30 arb, and auxiliary gas 10 arb. The capillary temperature was 325°C, the full scan was performed with a resolution of 60,000, the scanning range was 81∼1,000, the HCD was used for secondary cracking, the collision voltage was 30 eV, and the use of dynamic elimination to remove unnecessary MS/MS information.

Structures corresponding to the selected metabolites were obtained by searching the freely accessible HMDB^1^, KEGG^2^) databases, and self-built standard product database (Supplementary Table 3).

### FMT

Before transplantation, mice were treated for two consecutive weeks with 200 μl of an antibiotic cocktail (with each daily dose being administered by oral gavage after a 6-h fast) that contained 1 g/L ampicillin, 0.5 g/L neomycin, 0.5 g/L vancomycin, and 1 g/L metronidazole (Gonzalez et al., 2016). Thereafter, mice were given 200 μl of the microbiota suspension once a day for 4 weeks, starting the first day after the antibiotic cycle. After a 2-week period, mice received the microbiota suspension once a week until natural death or sacrifice. For the microbiota suspension preparation, two to five fresh feces pellets (80-100 mg) were resuspended with a vortex in 600 μl of reduced PBS (PBS with 0.5 g l-1cysteine and 0.2 g l-1Na_2_S). After resuspension, tubes containing the feces in reduced PBS were centrifuged at 2,500 r.p.m. (500 g) for 1 min to remove insolubilized material, and 100 μl of supernatant was administered to the mice by oral gavage. Mice in the empty transplant group received the same antibiotics treatment and were transplanted only with reduced PBS.

### *In vitro* fermentation experiments

The CON group (1 ml bacterial liquid and 9 ml MRS broth medium), the HFD group (1 ml bacterial liquid and 9 ml MRS broth medium), the LGG + CON group (1 ml bacterial liquid and 1 ml *Lactobacillus rhamnosus* GG liquid and 8 ml MRS broth medium), and the LGG + HFD group (1 ml bacterial liquid and 1 ml *L. rhamnosus* GG liquid and 8 ml MRS broth medium) were incubated in an anaerobic chamber with 5% CO_2_, 10% H_2_, and 85% N_2_ for 24 h, then placed it in an anaerobic bacteria incubator at 37°C for 18 h, took the supernatant of the fermentation broth, and detected the content of 5-MIAA by UPLC-MS/MS.

### Cell cultures

The HepG2 cells were obtained from our laboratory. The HepG2 cells were cultured in DMEM with 10% FBS at 37°C in a humidified 5% CO_2_ atmosphere. Cell passages were performed with 0.25% trypsin-EDTA (Gibco, Life Technologies) when cells reached 80 to 90% confluency and then divided into control group (CON), 5-methoxyindole acetic acid administration group (5-MIAA, 0.1025 g/100 μl) based on the previous study of Saeedi et al. (2020), and tert-butyl hydroquinone group (TB, 0.167 g/100 μl) based on the previous study (Nna et al., 2020).

### Statistical analysis

All data were shown as means ± standard deviation (SD). Data sets that involved more than two groups were assessed by one-way ANOVA followed by Newman–Keuls *post hoc* tests. *p* < 0.05 was considered statistically significant. For metabolomics analysis, data were normalized to the internal standard and all variables were pareto-scaled before analyses. R^3^ and Graphpad Prism 6.0 software (GraphPad, CA, United States) were used for statistical analysis and graphics.

## Results

### Preparation and quantitative profiling of Tianhuang formula

The main active components of *P. notoginseng* root and rhizome were ginsenoside Rg1, ginsenoside Rb1, ginsenoside Rd, ginsenoside Re, and *P. notoginseng* saponin R1. The major active components of *C. chinensis* root were berberine, coptisine, and bamatine. TH was made of ginsenoside Rg1 (23.82%), ginsenoside Rb1 (4.58%), ginsenoside Rd (0.97%), ginsenoside Re (1.03%), *P. notoginseng* saponin R1 (2.05%), coptisine (4.45%), bamatine (5.11%), berberine (17.01%), and some other unidentified components (41.98%) by HPLC analysis.

### High-fat diet-induced non-alcoholic fatty liver disease mice have disorders of the gut microbiome and lipid metabolism and hepatic stress response

**FIGURE 1:**
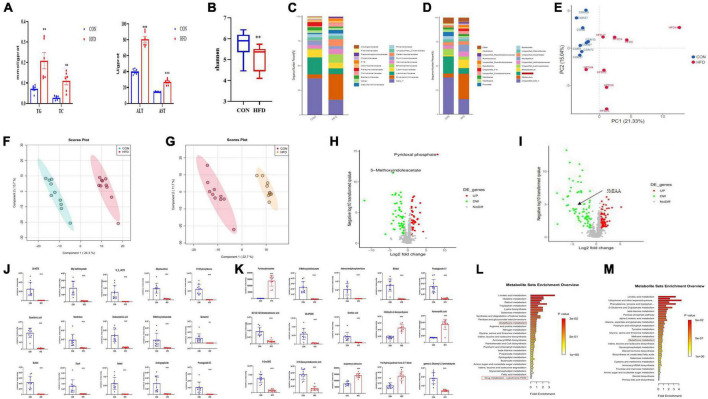
NAFLD induced by HFD with gut microbiome and metabolic disorders, oxidative stress in mice. **(A)** Hepatic TC, TG, AST, and ALT levels in mice. **(B)** Box-plot of the Shannon of the CON and HFD groups. **(C,D)** Analysis of the different relative abundances of the bacterial community at the family and genus levels in CON and HFD mice. **(E)** PCA score plot analysis based on the relative abundance of OTUs. Different color indicates each group of mice (*n* = 8), and each spot represented one mouse. **(F,G)** PLS-DA score plot analysis of metabolites in liver (R2Y = 0.937, Q2 = 0.89) and intestinal contents (R2Y = 0.942 Q2 = 0.879). **(H,I)** Volcano plot of metabolites enrichment analysis of liver tissue and intestinal contents in the control and model group. **(J)** The contents of the top 15 altered features identified from metabolites of liver tissue from the experiment described in **(H)**. **(K)** The contents of the top 15 altered features identified from UPLC-MS/MS analysis in intestinal contents from the experiment described in **(I)**. **(L,M)** Mummichog pathway analysis of liver tissue and intestinal contents for the identification of pathways induced in the HFD group relative to CON groups. Red boxes highlight pathways involved in antioxidant and xenobiotic metabolism. All the data were presented as the means ± SD from *n* = 8-10 mice per group. Significant differences compared with the CON group were indicated by asterisks: *P < 0.05, **P < 0.01, and ***P < 0.001.

The contents of hepatic TC and TG and serum ALT and AST in NAFLD mice induced by HFD increased significantly (Figure 1A). We compared the characteristics of the gut microbiome of NAFLD mice and found that the Shannon was decreased significantly (Figure 1B), and the gut microbiome was different at the level of the family, species, and genus, especially *Allobaculum* increased, while *lactobacillus* decreased significantly (Figures 1C-E). We speculated that NAFLD may be closely related to the decrease of *lactobacillus*.

We performed metabonomics of intestinal contents and liver tissue to find out the changes in metabolites in NAFLD mice. The results showed that metabolites were well separated from the PLS-DA figure both in the intestinal contents and liver tissue (Figures 1F-I), indicating that there were significant differences in the metabolites in the liver and intestinal contents between the CON group and the HFD group. The contents of the top 15 metabolites that have changed significantly among them were statistically analyzed and found that the metabolites such as pyridoxal phosphate and homovanillic acid increased significantly, while 5-Methoxyindoleacetate and adenosine diphosphate ribose decreased significantly. Metabolites such as pyridoxal phosphate, 5-Methoxyindoleacetate, and prostaglandin E1 in the liver were significantly reduced in NAFLD mice (Figures 1J,K). 5-Methoxyindoleacetate was significantly reduced both in intestinal contents and liver, so we speculated that it was closely related to NAFLD. To explore the correlation between metabolites and NAFLD, we conducted a pathway analysis and found that the enrichment pathways of metabolites in the intestinal contents and liver related to oxidative stress (Figures 1L,M). These data showed that NAFLD mice may induce hepatic oxidative stress.

### The feces of NAFLD mice induced by high-fat diet led to oxidative liver injury

**FIGURE 2:**
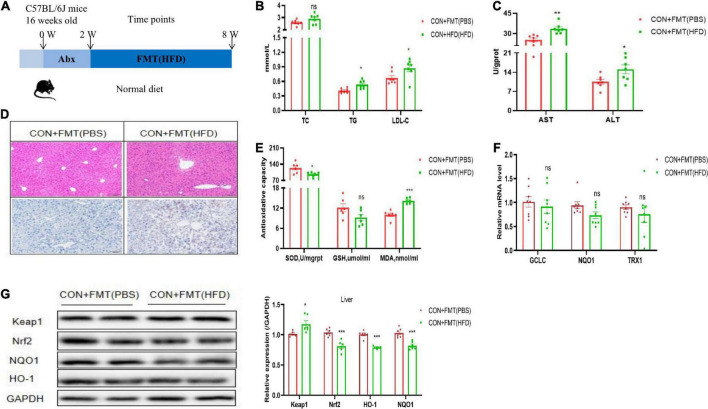
Transfer of FTM promoted oxidative liver injury. **(A)** Schematic of experimental design. **(B)** Representative H&E images and Oil red O staining of mice livers. **(C)** Serum TC, TG, and LDL-C of mice treated with vehicle or FTM. **(D)** Hepatic AST and ALT of mice treated with vehicle or FTM. **(E)** Hepatic SOD, MDA, and GSH of mice treated with vehicle or FTM. **(F)** Relative mRNA expression of the oxidative markers GCLC, NQO1, and TXR1 of mice treated with vehicle or FTM. **(G)** Western blot analyses of Keap1, Nrf2, NQO1, and HO-1. All the data were presented as the means ± SD from *n* = 8 mice per group. Significant differences compared with the CON + FMT(PBS) group were indicated by asterisks: *P < 0.05, **P < 0.01, and ***P < 0.001.

To further clarify the relationship between NAFLD induced by HFD and the gut microbiome, we conducted an FMT experiment. The flow chart of FMT is shown in Figure 2A. Before FMT, the CON group mice were gavaged with antibiotics (1 g/L ampicillin, 0.5 g/L neomycin, 0.5 g/L vancomycin, and 1 g/L metronidazole), and then the feces of the HFD mice were transplanted to the CON group, named CON + FMT (HFD) group, the transplantation time was 4 weeks, and other group named CON + FMT (PBS) group. The results showed that the content of serum TG and LDL-C increased significantly in the CON + FMT (HFD) group (Figure 2B). The serum ALT and AST levels in the CON + FMT (HFD) group increased significantly (Figure 2C). The CON + FMT (HFD) group mice had hepatic steatosis from the hepatic HE staining and oil red O staining (Figure 2D), and hepatic oxidative stress indicators such as the contents of hepatic GSH and SOD were reduced, and MDA increased significantly (Figure 2E). The mRNA expression of TRX1, GCLC, and NQO1 decreased (Figure 2F), the protein expression of Keap1 increased, and Nrf2, NQO1, and HO-1 decreased in the CON + FMT (HFD) group (Figure 2G), indicating that NAFLD induced by HFD closely related to the gut microbiome, and the oxidative liver injury mediated by the Nrf2 pathway played an important role in it.

### TH improved the oxidative stress response of NAFLD induced by high-fat diet

**FIGURE 3:**
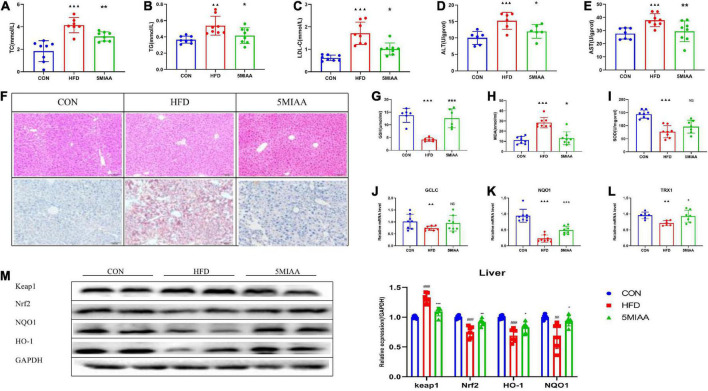
TH exhibited protection against NAFL. **(A–C)** Serum TC, TG, and LDL-C of HFD mice treated with TH (60 mg/kg) or AT (1.5 mg/kg). **(D,E)** Hepatic AST and ALT of HFD mice treated with TH or AT. **(F)** Representative H&E images and Oil red O staining of mouse livers. **(G–I)** Hepatic SOD, MDA, and GSH of mice treated with TH. **(J–L)** Relative mRNA expression of the oxidative markers GCLC, NQO1, and TXR1 of mice treated with TH. **(M)** Western blot analyses of Keap1, Nrf2, NQO1, and HO-1. All the data were presented as the means ± SD from *n* = 6-8 mice per group. Significant differences compared with the CON group were indicated by asterisks: ^▲^*P* < 0.05, ^▲▲^*P* < 0.01, ^▲▲▲^*P* < 0.001. Significant differences compared with the HFD are indicated by asterisks: *P < 0.05, **P < 0.01, and ***P < 0.001.

We further researched the effect of TH to improve NAFLD and found that TH reduced serum TC, TG, LDL-C, ALT, and AST significantly (Figure 3A-F), and improved hepatic steatosis of NAFLD mice. TH improved the hepatic oxidative stress response with the levels of hepatic GSH and SOD increasing and MDA reducing (Figures 3G-I). TH increased the mRNA expression of hepatic TRX1, GCLC, and NQO1 (Figures 3J-L), decreased the protein expression of Keap1, and increased Nrf2, NQO1, and HO-1 of NAFLD mice (Figure 3M), indicating that TH activated Nrf2 pathway, inhibited oxidative stress, and protected against NAFLD.

### TH-treated mice harbored a distinct gut microbiota profile that contributed to reducing NAFLD

**FIGURE 4:**
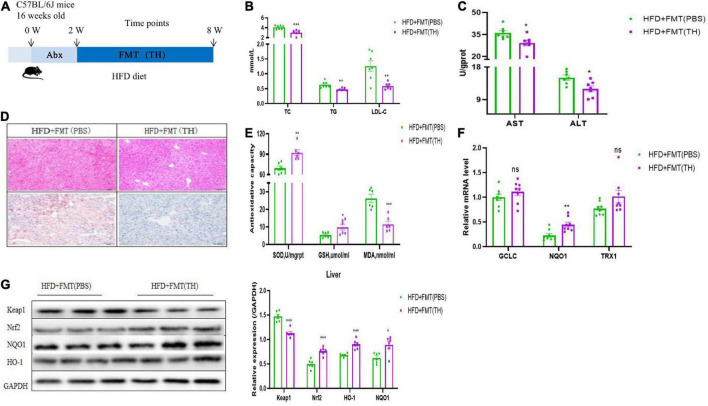
The gut microbiota alterations associated with Nrf2 activation contributed to the effects of TH. **(A)** Schematic of experimental design. **(B)** Representative H&E images and Oil red O staining of mice livers. **(C)** Serum TC, TG, and LDL-C in HFD + FMT(PBS) and HFD + FMT(TH) group. **(D)** Hepatic AST and ALT. **(E)** Hepatic SOD, MDA, and GSH. **(F)** Relative mRNA expression of the oxidative markers GCLC, NQO1, and TXR1. **(G)** Western blot analyses of Keap1, Nrf2, NQO1, and HO-1. All the data were presented as the means ± SD from n = 6-8 mice per group. Significant differences compared with the HFD + FMT(PBS) were indicated by asterisks: *P < 0.05, **P < 0.01, and ***P < 0.001.

To further clarify whether TH improved NAFLD by regulating the gut microbiota, we transplanted fecal of TH-treated mice to the HFD group, named HFD + FMT(TH) group. The flow chart of FMT is shown in Figure 4A. Before FMT, the HFD group mice were gavaged with antibiotics (1 g/L ampicillin, 0.5 g/L neomycin, 0.5 g/L vancomycin, and 1 g/L metronidazole), and then carried out FMT for 4 weeks. The results showed that the serum levels of TC, TG, and LDL-C (Figure 4B) and hepatic ALT and AST in HFD + FMT (TH) group reduced significantly (Figure 4C). The hepatic steatosis was improved from the hepatic HE staining and Oil Red O staining (Figure 4D). The feces of TH mice improved hepatic oxidative stress indicators, increased the levels of hepatic GSH and SOD, and decreased MDA (Figure 4E). The mRNA expression of hepatic TRX1, GCLC, and NQO1 increased (Figure 4F), the protein expression of Keap1 decreased, while Nrf2, NQO1, and HO-1 increased (Figure 4G) and the Nrf2 pathway was activated in the HFD + FMT (TH) group, indicating that TH improved NAFLD via regulating gut microbiota.

### The genus *Lactobacillus*-mediated generation of 5-MIAA was capable of activating hepatic Nrf2 signaling

**FIGURE 5:**
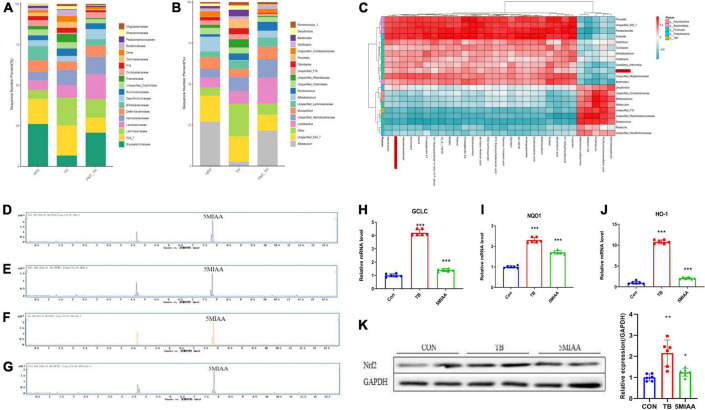
The genus *Lactobacillus* mediated generation of 5-MIAA was capable of activating hepatic Nrf2 signaling. **(A,B)** Analysis of the different relative abundances of the bacterial community at the family and genus levels in HFD, TH, and HFD + FMT(TH) groups. **(C)** The correlations between the relative abundance of the main genus of the bacterial community and the top 30 metabolites in the HFD and FMT (TH) groups. **(D–G)** Content of 5-MIAA analyzed by UPLC-MS/MS in the fermentation broth of CON, HFD, CON + LGG, and HFD + LGG groups. **(H–J)** Relative mRNA expression of the GCLC, NQO1, and TXR1 of HepG2 cells treated with 5-MIAA. **(K)** Western blot analyses of Nrf2 in HepG2 cells treated with 5MIAA. All the data were presented as the means ± SD from n = 6 mice per group. Significant differences compared with the CON group were indicated by asterisks: *P < 0.05, **P < 0.01, and ***P < 0.001.

We found that the *Lactobacillus* in NAFLD mice were reduced significantly, but whether the improvement of TH on NAFLD was related to *Lactobacillus* needs to be revealed. To determine the microbial populations responsible for the observed activation of hepatic Nrf2, we detected the abundance of *Lactobacillus* in the TH group and HFD + FMT (TH) group. Our results revealed that members of the genus *Lactobacillus* increased significantly both in the TH group and HFD + FMT (TH) group (Figure 5A-B). We analyzed the correlation between the top 30 significantly changed hepatic metabolites and the gut microbiota of NAFLD mice and found that 5-MIAA was positively correlated with *Lactobacillus* (Figure 5C). To further prove the relationship between the *Lactobacillus* and 5-MIAA, the feces of mice in the CON group and the HFD group were fermented *in vitro*, the content of 5-MIAA was measured in the fermentation broth by UPLC-MS/MS and found that after adding *L. rhamnosus GG(*LGG*)*, 5-MIAA increased in the fermentation broth (CON group, 5-MIAA = 0.96394 μg/ml; HFD group, 5-MIAA = 0.62085 μg/ml; LGG + CON group, 5-MIAA = 1.221209 μg/ml; LGG + HFD group, 5-MIAA = 0.87939 μg/ml), indicating 5-MIAA produced by *Lactobacillus* (Figures 5D-G).

Next, to discover whether the 5-MIAA were capable of activating hepatic Nrf2 signaling, the HepG2 cells were used to verify the effect of the 5-MIAA on oxidative stress-related gene expression and Nrf2 protein expression and found that 5-MIAA reduced the mRNA expression of GCLC, HO-1, and NQO1 and increased protein expression of Nrf2 significantly, which was consistent with the strong antioxidant tert-Butylhydroquinone (TB) (Figures 5H-K). It showed that TH protected against oxidative liver injury of NAFLD by adjusting the genus *Lactobacillus* mediated generation of 5-MIAA which were capable of activating hepatic Nrf2 signaling.

### 5-MIAA improved the oxidative stress response of NAFLD mice induced by high-fat diet

**FIGURE 6:**
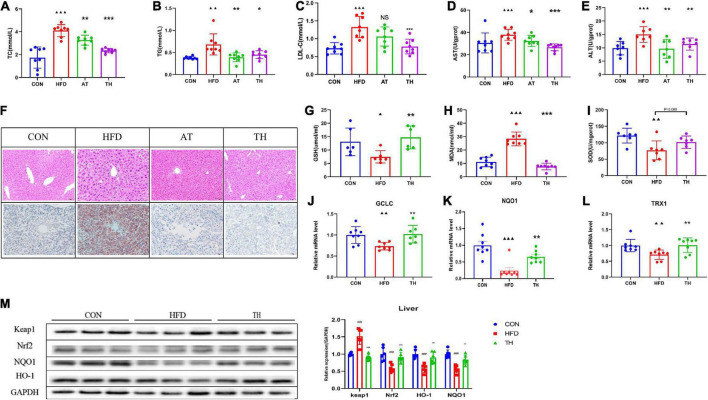
The genus *Lactobacillus*-mediated generation of 5-MIAA improved NAFLD. **(A–C)** Serum TC, TG, and LDL-C in NAFLD mice treated with vehicle or 5-MIAA (2.8 mM/kg). **(D,E)** Hepatic ALT and AST treated with vehicle or 5-MIAA (2.8 mM/kg). **(F)** H&E-stained sections and Oil red O staining of liver tissue of the CON, HFD, and 5-MIAA groups. **(G–I)** Hepatic SOD, MDA, and GSH. **(J–L)** Relative mRNA expression of GCLC, NQO1, and TXR1. **(M)** Western blot analyses of Keap1, Nrf2, NQO1, and HO-1. All the data were presented as the means ± SD from *n* = 6-8 mice per group. Significant differences compared with the CON group were indicated by asterisks: ^▲^*P* < 0.05, ^▲▲^*P* < 0.01, ^▲▲▲^*P* < 0.001. Significant differences compared with the HFD group were indicated by asterisks: *P < 0.05, **P < 0.01, and ***P < 0.001.

To further prove the improvement effect of 5-MIAA on NAFLD, we gavaged 5-MIAA to NAFLD mice induced by HFD for 8 weeks, and the results showed that 5-MIAA reduced the levels of serum TC, TG, and LDL-C (Figures 6A-C), hepatic ALT and AST (Figures 6D,E), and improved hepatic steatosis of NAFLD mice (Figure 6F). The 5-MIAA improved the hepatic oxidative stress response, increased the levels of hepatic GSH and SOD, and reduced MDA (Figures 6G-I), and also increased the mRNA expression of TRX1, GCLC, and NQO1 (Figures 6J-L), reduced the protein expression of Keap1, increased Nrf2, NQO1, and HO-1 (Figure 6M), and activated the Nrf2 pathway. The 5-MIAA is a novel *Lactobacillus*-derived small molecule capable of activating hepatic Nrf2.

## Discussion

Epidemiological studies have shown that NAFLD has become one of the most widespread chronic hepatic diseases in the world. The median prevalence of NAFLD is about 20%, with a progressively increasing trend (Younossi et al., 2011). The treatment of NAFLD currently mainly includes the following three aspects: (a) Lifestyle change to promote weight loss through diet and physical activity, (b) control of the main cardiometabolic risk factors, and (c) correction of all modifiable risk factors leading the development and progression of advanced forms of NAFLD, and prevention of hepatic and extra-hepatic complications (Mantovani and Dalbeni, 2021). Because the pathogenesis of NAFLD has not been well understood, there is still no effective drug to prevent and treat NAFLD (Lin and Kohli, 2020).

NAFLD belongs to the theory of traditional Chinese medicine “Danzhuo,” which was first put forward by professor Guo Jiao from Guangdong pharmaceutical university and found that oxidative stress is a vital basis for NAFLD (Specialty Committee of Metabolic Diseases of WFCMS, 2019). Chinese medicines have been proven to have the effect of anti-inflammatory, antioxidant, and lipid homeostasis, and improving insulin sensitivity may be a potential strategy for NAFLD.

TH is mainly composed of *P. Notoginseng* and *Rhizoma Coptidis*, and the effects of its main components on NAFLD have been reported. *P. notoginseng saponins* mediate the gut-liver axis to improve hepatic steatosis and fibrosis in NAFLD mice via a TLR4-dependent manner (Xu et al., 2021). Berberine (isoquinoline alkaloid) increases mitochondrial SIRT3 activity and improves hepatic OXPHOS in a high-fat diet induced rats (Teodoro et al., 2013). Our previous research showed that TH has a lipid-lowering effect (Li K. P. et al., 2021) and low drug permeability, which results in poor intestinal absorption into the human body, and was thus able to come into contact with the gut microflora for a longer time which might be able to influence the gut microbial ecosystem, but it still needs to be investigated.

As an indispensable part of the human body, the gut microbiome has played an important role in NAFLD, obesity, and lipid metabolism disorders. The relationship between NAFLD and the gut microbiome has become a hotspot of current research (Gonzalez et al., 2016). López-Almela’s studies find that the combined treatment of *B. uniformis* and wheat bran extract reduces the body weight, TC, and blood glucose in diet-induced obese mice, which relates to the anti-inflammatory properties of butyrate (López-Almela et al., 2021). Indole, as a bacterial metabolite of tryptophan, has the effect of directly reducing liver injury and relates to inflammatory response and macrophage activation in ob/ob mice (Knudsen et al., 2021). Citrulline improves NAFLD by inhibiting the translocation of gut microbial, reducing the loss of tight junction proteins, and increasing the activity of small intestinal arginase (Rajcic et al., 2021). The composition and metabolites of the gut microbiota have also become key factors that regulate the pathological process of NAFLD. Key metabolites produced by the gut microbiota, including short-chain fatty acids, secondary bile acids, indole and its derivatives, trimethylamine, carotenoids, and phenolic compounds, play a regulatory role in host metabolism, immune cell system, and redox homeostasis, thereby fundamentally changing the progress of NAFLD (Ji et al., 2019). Although many studies have suggested the interaction between gut microbes and NAFLD, the underlying mechanism has not yet been fully elucidated.

In this study, we found that the abundance of gut microbiota of NAFLD mice induced by HFD was significantly different from those of normal diet mice such as *Unspecified_S24_7*, *Allobaculum*, *Lactobacillus*, and *Bifidobacterium*. The metabolites such as pyridoxal phosphate, 5-methoxyindoleacetate, prostaglandin E1, and so on changed significantly in intestinal contents and hepatic tissue. We selected the main hepatic metabolites and the gut microbiota for correlation analysis, and found that *Lactobacillus* was positively correlated with 5-MIAA, *Lactobacillus*-mediated generation of 5-MIAA by fermentation experiments *in vitro*, and 5-MIAA as a novel small molecule capable of activating hepatic Nrf2.

*L. rhamnosus GG* is a general term for a class of non-spore-free, Gram-stain-positive bacteria whose main product is lactic acid, mainly including *L. rhamnosus*, *Lactobacillus plantarum*, *Lactobacillus acidophilus*, and so on. As a beneficial flora that settles in the intestinal tract, it has a variety of physiological functions. Studies have found that colonizing the rat model of acute liver injury with *Lactobacillus casei Daitian strain, LcS* affected the composition of the gut microbiome, metabolism, and regulation of transcription, and significantly alleviate the liver injury phenotype (Yan et al., 2021). *L. plantarum* has been revealed to be effective in ameliorating NAFLD (Ritze et al., 2014). Zhao et al. (2020) reported that *L. plantarum NA136* improves NAFLD in a mouse model induced by a high-fat/high-fructose diet (Zhao et al., 2020). This study found that *Lactobacillus* and its related metabolite 5-MIAA significantly reduced in the NAFLD. The feces treated with TH increased the abundance of *Lactobacillus* in NAFLD mice by FMT.

Hepatic steatosis and lipid accumulation are the key pathological features of NAFLD, as well as the key factors leading to a subsequent decline in liver function and liver damage (Lebeaupin et al., 2018). This study showed that TH reduced the levels of serum and hepatic TC, TG, and LDL-C in NAFLD mice, and reduced hepatic steatosis and lipid accumulation. Hepatic injury and dysfunction are important driving factors for the progression of hepatic disease, including NAFLD. ALT and AST are important indicators for evaluating liver injury and liver function in NAFLD. A long-term high-fat diet caused liver injury, decreased liver function, and increased levels of ALT and AST (Kwo et al., 2017). This study has shown that TH reduced the levels of hepatic ALT and AST in NAFLD mice induced by HFD significantly, suggesting that TH protects against liver injury. We also found that TH improved the levels of hepatic MDA, GSH, and SOD, improved the oxidative stress response of NAFLD mice by activating the Nrf2 pathway, and further found that TH and feces treated with TH had a similar effect on NAFLD.

In this study, we found that obvious lipid droplets appeared in the liver in NAFLD mice induced by HFD after 12 weeks and densely appeared after 24 weeks, accompanied by a significant increase of serum TC, TG, and LDL-C, hepatic AST, ALT, and MDA, and decrease of hepatic GSH and SOD. The metabolites and gut microbiota were changed significantly. TH increased the content of *Lactobacillus* and its metabolite 5-MIAA. FMT verified that TH’s effect on improving NAFLD was related to the gut microbiota. We revealed that TH improved NAFLD via the “Lactobacillus-5-MIAA-Nrf2” pathway.

In summary, these findings add to our understanding of the functional consequences of the microbiome-gut-liver axis. Furthermore, we established *Lactobacillus* as potent drivers of hepatic Nrf2 and added to the significant body of literature supporting its potential as a hepatoprotective agent. These data also indicated that Chinese medicine improved NAFLD by regulating gut microbiota may be an effective strategy.

## Limitations of study

While we demonstrate in this study that *Lactobacillus* was capable of activating Nrf2 at a distance via the production of the small molecule 5-MIAA, several key limitations should be noted. First, we only tested a few representative microbes for systemic Nrf2 activation in the mice model. These microbes do not cover the gut microbiome as a whole, and it would be interesting to determine what other bacterial taxa have this capability. Another limitation of this study was that analysis of metabolites in intestinal contents and hepatic tissue from NAFLD mice revealed 260 differentially abundant features, of which only 5-MIAA was identified as a hepatoprotective agent. It will be interesting to identify those other small molecules and characterize their NAFLD improving capacity.

## Data availability statement

The datasets presented in this study can be found in online repositories. The names of the repository/repositories and accession number(s) can be found in the article/Supplementary material.

## Ethics statement

The animal study was reviewed and approved by Animal Ethical Committee of Guangdong Pharmaceutical University.

## Author contributions

JG and DL were responsible for the conception and design of the study. LY, HP, YZ, KL, and XR for the data collection, analysis, image processing, and writing the manuscript. All authors read and approved the final manuscript.

## Footnotes

1
http://www.hmdb.ca

2
http://www.genome.jp

3
https://www.r-project.org

## Abbreviations

NAFLD: non-alcoholic fatty liver disease
qRT-PCR: quantitative real-time PCR
FMT: fecal microbiota transplantation
5-MIAA: 5-Methoxyindoleacetate
GSH: glutathione
SOD: superoxide dismutase
MDA: malondialdehyde
Nrf2: Nuclear factor erythroid 2 related factor 2
NQO1: Recombinant NADH Dehydrogenase Quinone 1
HO-1: heme oxygenase 1
HFD: High-fat diet
HPLC: High-Performance Liquid Chromatography
UPLC: Ultra Performance Liquid Chromatography
AT: Atorvastatin
PBS: phosphate-buffered saline
DMEM: dulbecco’s modified eagle medium
TG: triglycerides
TC: total cholesterol
LDL-C: lipoprotein cholesterol
ALT: alanine transferase
AST: aspartate transferase
H&E: hematoxylin and eosin
TRX1: Thioredoxin-1
GCLC: Recombinant Glutamate Cysteine LigaseCatalytic
TB: tert-Butylhydroquinone
LGG: *Lactobacillus rhamnosus GG;*
TH: Tianhuang formula.
