# Supplementary material for: Tianhuang formula reduces the oxidative stress response of NAFLD by regulating the gut microbiome in mice
Source: Front Microbiol. 2022 Sep 21;13:984019. doi: 10.3389/fmicb.2022.984019 (PMC9533869; doi:10.3389/fmicb.2022.984019)
Supplement: Supplementary file 1 [file Data_Sheet_1.docx]

Table S1. Primer sequences of genes for qPCR.

| Genes | Forward (from 5’to 3’) | Reverse (from 5’to 3’) |
| --- | --- | --- |
| GAPDH | GCATCCACTGGTGCTGCC | TCATCATACTTGGCAGGTTTC |
| GCLC | GGGGCTGCTGTCCCAA | CAACATGTACTCCACCTCGTC |
| HO-1 | AGTTCAAGCAGCTCTACCGC | GCAACTCCTCAAAGAGCTGGAT |
| NQO1 | GGTGAGCTGAAGGACTCGAA | CCACTGCAATGGGAACTGAAAT |
| TRX1 | AGCTTGTCGTGGTGGACTTC | AGTCTGCAGCAACATCCTGG |

Table S2. List of Antibodies

| Antibody | Supplier | Cat. No. | Host |
| --- | --- | --- | --- |
| Keap1 | Abcam | ab227828 | Rabbit |
| NQO1 | Abcam | ab28947 | Mouse |
| HO-1 | Abcam | ab189491 | Rabbit |
| Nrf2 | Abcam | ab137550 | Rabbit |
| GAPDH | Abcam | ab8245 | Mouse |

Table S3 The differentially metabolites of hepatic tissue in mice with HFD-induced NAFLD

| **NO** | **Metabolites** | **VIP** | **p.value**  **p.value** |
| --- | --- | --- | --- |
| 1 | 20-HETE | 2.082345347 | 5.84E-14 |
| 2 | Allyl isothiocyanate | 2.060160471 | 7.69E-12 |
| 3 | 19_S_-HETE | 2.058195531 | 4.27E-12 |
| 4 | Dihydrocortisol | 2.043251412 | 6.68E-11 |
| 5 | 57-Dihydroxyflavone | 2.037097927 | 1.29E-09 |
| 6 | Stearidonic acid | 2.029138697 | 3.73E-09 |
| 7 | Nandrolone | 2.005475274 | 5.29E-10 |
| 8 | Dodecanedioic acid | 2.002955861 | 4.33E-09 |
| 9 | 5-Methoxyindoleacetate | 1.989391418 | 9.76E-08 |
| 10 | Quinestrol | 1.988943588 | 2.00E-07 |
| 11 | Bufalin | 1.986625712 | 9.84E-08 |
| 12 | Equol | 1.983001703 | 9.29E-08 |
| 13 | Sotalol | 1.980780342 | 2.64E-09 |
| 14 | Andrographolide | 1.976725402 | 1.55E-07 |
| 15 | Prostaglandin E3 | 1.946374196 | 2.84E-08 |
| 16 | Hexadecanedioate | 1.927213291 | 1.00E-07 |
| 17 | 7a-Hydroxyandrost-4-ene-317-dione | 1.903018091 | 7.25E-07 |
| 18 | Prostaglandin E1 | 1.901555694 | 1.24E-07 |
| 19 | Losartan | 1.895414077 | 1.69E-06 |
| 20 | Methaqualone | 1.892011579 | 1.58E-07 |
| 21 | Retinal | 1.867641479 | 5.33E-07 |
| 22 | all-trans-Retinoic acid | 1.861619921 | 9.30E-06 |
| 23 | Glycitein | 1.843592792 | 1.26E-05 |
| 24 | Methionine sulfoximine | 1.843287212 | 7.59E-07 |
| 25 | Aflatoxin G2 | 1.834667272 | 2.66E-05 |
| 26 | 910-Epoxyoctadecenoic acid | 1.830900974 | 2.58E-06 |
| 27 | Prednisone | 1.811783604 | 1.86E-06 |
| 28 | Perfluorooctanesulfonic acid | 1.793489165 | 3.49E-06 |
| 29 | Normetanephrine | 1.791598702 | 7.13E-06 |
| 30 | Gamma-Linolenic acid | 1.773673816 | 5.03E-06 |
| 31 | dTMP | 1.76271216 | 0.00010551 |
| 32 | Calcitriol | 1.758193565 | 4.77E-05 |
| 33 | 5_S_-HpETE | 1.757416387 | 8.85E-06 |
| 34 | 4-Hydroxyphenylpyruvic acid | 1.748825697 | 1.04E-05 |
| 35 | Midodrine | 1.742036026 | 1.29E-05 |
| 36 | 11-Dehydro-thromboxane B2 | 1.729163793 | 8.29E-05 |
| 37 | Deoxycytidine | 1.729117631 | 3.23E-05 |
| 38 | Sarsasapogenin | 1.728655939 | 8.69E-05 |
| 39 | Abietate | 1.728025801 | 3.35E-05 |
| 40 | 11-Dehydrocorticosterone | 1.726584147 | 1.52E-05 |
| 41 | Dehydroepiandrosterone | 1.722127906 | 2.30E-05 |
| 42 | 9-OxoODE | 1.720139993 | 1.85E-05 |
| 43 | 13_S_-HpOTrE | 1.709406693 | 0.00014607 |
| 44 | Retinoyl b-glucuronide | 1.704853113 | 5.30E-05 |
| 45 | Cortisol 21-acetate | 1.699829647 | 0.00026174 |
| 46 | CDP | 1.695417174 | 3.10E-05 |
| 47 | Sorbitol 6-phosphate | 1.695138501 | 0.00013543 |
| 48 | Jasmonic acid | 1.69264107 | 6.26E-05 |
| 49 | gamma-L-Glutamyl-L-2-aminobutyrate | 1.691427342 | 0.00011302 |
| 50 | Naringenin | 1.686062776 | 0.00023709 |
| 51 | myo-Inositol | 1.685331319 | 3.96E-05 |
| 52 | Myristoleic acid | 1.67721686 | 0.00026466 |
| 53 | Docosapentaenoic acid _22n-3_ | 1.675578351 | 5.19E-05 |
| 54 | Glycyrrhetinate | 1.671767303 | 5.90E-05 |
| 55 | Homovanillic acid | 1.66923892 | 4.38E-05 |
| 56 | Digalacturonate | 1.662560554 | 5.31E-05 |
| 57 | Melatonin | 1.661792715 | 0.00014654 |
| 58 | 34-Dihydroxyphenylglycol | 1.650777076 | 5.72E-05 |
| 59 | Allocystathionine | 1.628990195 | 0.00010453 |
| 60 | L-Fucose | 1.624495779 | 0.00011888 |
| 61 | Tetrahydrocortisone | 1.621310215 | 0.00067544 |
| 62 | Protoporphyrin IX | 1.612905466 | 0.00035826 |
| 63 | Methylisoeugenol | 1.60141755 | 0.00066698 |
| 64 | L-Glutamic acid | 1.596595952 | 0.00020542 |
| 65 | L-Serine | 1.589128879 | 0.00019182 |
| 66 | Adenylsuccinic acid | 1.585360683 | 0.000242 |
| 67 | Hepoxilin B3 | 1.584608041 | 0.00018461 |
| 68 | Dimethylglycine | 1.580930267 | 0.00029146 |
| 69 | 3S5S_-Carbapenam-3-carboxylic acid | 1.579629705 | 0.00021218 |
| 70 | Ritodrine | 1.575830384 | 0.00018498 |
| 71 | Lovastatin | 1.574270916 | 0.00034429 |
| 72 | Porphobilinogen | 1.564577056 | 0.00024652 |
| 73 | 3-Hydroxyanthranilic acid | 1.562648955 | 0.00022353 |
| 74 | alpha-D-Galactosyl-13-beta-D-galactosyl-14-N-acetyl-D-glucosamine | 1.561762721 | 0.00029946 |
| 75 | 3-Methylindole | 1.561719131 | 0.00022711 |
| 76 | 5-Methylthioadenosine | 1.554119024 | 0.00032246 |
| 77 | Nitrendipine | 1.547821917 | 0.00033663 |
| 78 | 2R_-2-Hydroxy-3-_phosphonatooxy_propanoate | 1.543262423 | 0.00031538 |
| 79 | Pipecolic acid | 1.527221776 | 0.0003627 |
| 80 | Lumichrome | 1.517796813 | 0.00040769 |
| 81 | UDP | 1.5114386 | 0.0005388 |
| 82 | Benazepril | 1.509605391 | 0.0015537 |
| 83 | Deoxycorticosterone acetate | 1.50453014 | 0.001383 |
| 84 | Spermidine | 1.500775729 | 0.00051179 |
| 85 | Oxoglutaric acid | 1.49845588 | 0.00072502 |
| 86 | Rosmarinic acid | 1.497873933 | 0.00052408 |
| 87 | 13S-hydroxyoctadecadienoic acid | 1.497591965 | 0.00061384 |
| 88 | Caffeate | 1.496806246 | 0.00076476 |
| 89 | 3-Ketosphingosine | 1.48918109 | 0.0016599 |
| 90 | Estradiol | 1.486899697 | 0.00086919 |
| 91 | _13E_-11a-Hydroxy-915-dioxoprost-13-enoic acid | 1.484767656 | 0.00064958 |
| 92 | Secalciferol | 1.476035291 | 0.0018664 |
| 93 | Phenylephrine | 1.472775085 | 0.00071119 |
| 94 | Ecdysone | 1.469876476 | 0.0010793 |
| 95 | 1112-DiHETrE | 1.468170384 | 0.0014335 |
| 96 | 1-Aminocyclopropanecarboxylic acid | 1.466522307 | 0.00087847 |
| 97 | N2-Succinyl-L-ornithine | 1.461336179 | 0.00094695 |
| 98 9898 | 3-Dehydro-2-deoxyecdysone | 1.459872619 | 0.00086715 |
| 99 | Qing Hau Sau | 1.457687655 | 0.0019749 |
| 10000 | 4-Guanidinobutanoic acid | 1.454804943 | 0.0017545 |
| 101 | Ascorbate | 1.452183263 | 0.0019519 |
| 102 | Lipoxin A4 | 1.450217823 | 0.0010055 |
| 103 | Probucol | 1.448890951 | 0.00094539 |
| 104 | 2-Hydroxy-6-pentadecylbenzoic acid | 1.446944606 | 0.0019788 |
| 105 | Ethyl icosapentate | 1.443688728 | 0.0021675 |
| 106 | D-4-Phosphopantothenate | 1.442416522 | 0.0010112 |
| 107 | N-Acetyl-D-glucosamine | 1.442366282 | 0.0010186 |
| 108 | Aminomalonic acid | 1.434639343 | 0.0013107 |
| 109 | Methyl hexadecanoic acid | 1.434189723 | 0.0011288 |
| 110 | Thioguanine | 1.430077923 | 0.0015836 |
| 111 | N-Acetyllactosamine | 1.427499865 | 0.0023853 |
| 112 | L-Threonine | 1.426323223 | 0.0012518 |
| 113 | Adrenosterone | 1.415374065 | 0.001357 |
| 114 | Genistein | 1.412254414 | 0.0015211 |
| 115 | 23-Cyclic CMP | 1.407443038 | 0.0018771 |
| 116 | Topiramate | 1.406642242 | 0.0015866 |
| 117 | Sphinganine 1-phosphate | 1.406219643 | 0.0016284 |
| 118 | Thymidine | 1.404244542 | 0.0021325 |
| 119 | Cortisone | 1.399648517 | 0.0019021 |
| 120 | 5-S-Methyl-5-thioinosine | 1.397348897 | 0.002469 |
| 121 | Nonadecanoic acid | 1.391218213 | 0.002021 |
| 122 | dAMP | 1.388658945 | 0.0017998 |
| 123 | N-Acetyl-D-tryptophan | 1.387418601 | 0.0018155 |
| 124 | 91213-TriHOME | 1.385853982 | 0.0020772 |
| 125 | Guanine | 1.38527947 | 0.0019478 |
| 126 | N-Acetylneuraminic acid | 1.382435285 | 0.0031522 |
| 127 | N-Acetyl-beta-alanine | 1.379739322 | 0.0039988 |
| 128 | Adenine | 1.370873646 | 0.0029845 |
| 129 | Neoabietic acid | 1.37081022 | 0.0022948 |
| 130 | Moxonidine | 1.367865425 | 0.0024889 |
| 131 | Methyleugenol | 1.365407746 | 0.003303 |
| 132 | Cybutryne | 1.361568405 | 0.0023988 |
| 133 | gamma-Glutamylalanine | 1.360766163 | 0.0023693 |
| 134 | Enoxacin | 1.35982574 | 0.0041019 |
| 135 | Sedoheptulose | 1.352651046 | 0.0025837 |
| 136 | 3-Methyl-L-tyrosine | 1.344772732 | 0.0043984 |
| 137 | N-Acetylleucine | 1.342369744 | 0.0028351 |
| 138 | Telmisartan | 1.339420979 | 0.0029771 |
| 139 | Ginkgolide B | 1.335122418 | 0.0056813 |
| 140 | Spectinomycin | 1.329051957 | 0.0035533 |
| 141 | 5-Hydroxyindoleacetic acid | 1.326931542 | 0.0033288 |
| 142 | Acetylphosphate | 1.315539259 | 0.0036738 |
| 143 | Glucose 6-phosphate | 1.313532875 | 0.0039034 |
| 144 | Xanthyletin | 1.307782759 | 0.0039309 |
| 145 | Ergosta-572224_28_-tetraen-3beta-ol | 1.304840143 | 0.0041185 |
| 146 | D-Xylose | 1.303346994 | 0.0046675 |
| 147 | Cytosine | 1.290231343 | 0.0069225 |
| 148 | Norcodeine | 1.288965706 | 0.0046294 |
| 149 | Sodium deoxycholate | 1.288499322 | 0.0046498 |
| 150 | 23-Butanediol | 1.286513526 | 0.0047065 |
| 151 | Dibutyl phthalate | 1.285841048 | 0.006749 |
| 152 | 17a-Estradiol | 1.276136178 | 0.0054463 |
| 153 | L-2-Aminoadipate adenylate | 1.274129387 | 0.0055555 |
| 154 | UMP | 1.273513351 | 0.0060977 |
| 155 | Nicotinamide riboside | 1.273404816 | 0.0077658 |
| 156 | Dehydroascorbate | 1.266944422 | 0.0057284 |
| 157 | Urocanic acid | 1.265880415 | 0.0077707 |
| 158 | Butyryl-L-carnitine | 1.265355313 | 0.0056386 |
| 159 | L-Erythrulose | 1.264883112 | 0.0066751 |
| 160 | FAPy-adenine | 1.263182627 | 0.0058075 |
| 161 | N2-gamma-Glutamylglutamine | 1.261392927 | 0.0064504 |
| 162 | Benzaldehyde | 1.259235905 | 0.011345 |
| 163 | Dihydrouracil | 1.259145973 | 0.0062389 |
| 164 | Phenylacetic acid | 1.256930194 | 0.0061697 |
| 165 | Gentisic acid | 1.254260409 | 0.0061809 |
| 166 | 11b-Hydroxyandrost-4-ene-317-dione | 1.24887634 | 0.0081027 |
| 167 | Carvedilol | 1.248572428 | 0.0073464 |
| 168 | 2-Deoxystreptamine | 1.247271766 | 0.01221 |
| 169 | Retinol | 1.239344782 | 0.0070159 |
| 170 | Guanosine | 1.234541426 | 0.0081174 |
| 171 | Zeranol | 1.233429991 | 0.010272 |
| 172 | all-trans-56-Epoxyretinoic acid | 1.230029899 | 0.0075728 |
| 173 | Acetylcholine | 1.228560601 | 0.0088009 |
| 174 | Maltol | 1.228444328 | 0.0076544 |
| 175 | N-Acetyl-L-phenylalanine | 1.221732948 | 0.00805 |
| 176 | Lincomycin | 1.221672235 | 0.0083231 |
| 177 | Acetohexamide | 1.217756261 | 0.0083467 |
| 178 | Carnosine | 1.216416314 | 0.008594 |
| 179 | Prostaglandin-c2 | 1.21144743 | 0.010178 |
| 180 | Biliverdin | 1.20972287 | 0.008918 |
| 181 | Vaccenic acid | 1.209193025 | 0.010187 |
| 182 | Fructose 16-bisphosphate | 1.209144186 | 0.010315 |
| 183 | Deoxycholic acid | 1.206147168 | 0.015563 |
| 184 | Saccharopine | 1.20548091 | 0.010772 |
| 185 | 25-Hydroxycholesterol | 1.198645918 | 0.0096372 |
| 186 | ciscis-Muconate | 1.198640089 | 0.011246 |
| 187 | Malvidin 3-glucoside | 1.194726765 | 0.0099429 |
| 188 | Nitrazepam | 1.19280811 | 0.010157 |
| 189 | 9Z12Z15Z_-Octadecatrienoic acid | 1.186609996 | 0.010609 |
| 190 | 1-Methylhistidine | 1.18557299 | 0.013632 |
| 191 | Aflatoxin B1 | 1.184203277 | 0.011272 |
| 192 | AMP | 1.18372709 | 0.012653 |
| 193 | Fucose 1-phosphate | 1.18038969 | 0.013523 |
| 194 | beta-D-Glucosamine | 1.172597545 | 0.012067 |
| 195 | 15-Deoxy-d-1214-PGJ2 | 1.169108529 | 0.013811 |
| 196 | Taraxerol | 1.159911675 | 0.01491 |
| 197 | Triacetate lactone | 1.151669113 | 0.014363 |
| 198 | 12-Hydroxydodecanoic acid | 1.148662117 | 0.014979 |
| 199 | alpha-Tocopherol | 1.145416983 | 0.017432 |
| 200 | L-Aspartic acid | 1.141496891 | 0.014648 |
| 201 | Flavonol 3-O-D-galactoside | 1.138845061 | 0.014945 |
| 202 | Vitamin D3 | 1.134788639 | 0.019715 |
| 203 | 10E12Z-Octadecadienoic acid | 1.130950597 | 0.019512 |
| 204 | 16-Hydroxy hexadecanoic acid | 1.125396316 | 0.018201 |
| 205 | Maleic acid | 1.122032303 | 0.016785 |
| 206 | Metoclopramide | 1.119422668 | 0.017139 |
| 207 | _S_-Reticuline | 1.118307467 | 0.017242 |
| 208 | 1-Arachidonoylglycerol | 1.112882475 | 0.018672 |
| 209 | 123-Trihydroxybenzene | 1.108897302 | 0.018344 |
| 210 | Methotrexate | 1.10789255 | 0.018795 |
| 211 | D-Arabinose | 1.107182451 | 0.019171 |
| 212 | 6-Phosphonoglucono-D-lactone | 1.10424952 | 0.021406 |
| 213 | 2-Pyrrolidinone | 1.103783349 | 0.021915 |
| 214 | Chavicol | 1.101157695 | 0.029219 |
| 215 | Phenyl acetate | 1.100400096 | 0.020557 |
| 216 | Estrone | 1.097717047 | 0.019828 |
| 217 | Choline | 1.094202603 | 0.02209 |
| 218 | L-Tyrosine | 1.092327636 | 0.02071 |
| 219 | L-Leucine | 1.086745394 | 0.022742 |
| 220 | Uracil | 1.081828591 | 0.024254 |
| 221 | 6-Phosphogluconic acid | 1.081271651 | 0.022476 |
| 222 | Pantetheine 4-phosphate | 1.078912675 | 0.025863 |
| 223 | Mefenamic acid | 1.076711892 | 0.023504 |
| 224 | Leukotriene E4 | 1.074222028 | 0.023066 |
| 225 | 1213-DHOME | 1.07274239 | 0.023984 |
| 226 | N6-_delta2-Isopentenyl_-adenosine 5-monophosphate | 1.071986222 | 0.024599 |
| 227 | Nicotinate D-ribonucleoside | 1.070706762 | 0.026066 |
| 228 | Niacinamide | 1.064155782 | 0.025937 |
| 229 | Vitexin | 1.061841315 | 0.025186 |
| 230 | Methyldopa | 1.061570592 | 0.025095 |
| 231 | Resolvin D2 | 1.057579454 | 0.02908 |
| 232 | 910-DHOME | 1.054171327 | 0.034277 |
| 233 | Arbutin | 1.053833936 | 0.026867 |
| 234 | 9-Riburonosyladenine | 1.050543464 | 0.027643 |
| 235 | Cyclic AMP | 1.049721569 | 0.033264 |
| 236 | Allantoin | 1.049416424 | 0.027291 |
| 237 | Norselegiline | 1.047276348 | 0.027347 |
| 238 | Deoxyinosine | 1.044829099 | 0.027827 |
| 239 | Delta-12-Prostaglandin J2 | 1.035437548 | 0.029565 |
| 240 | Citicoline | 1.034970249 | 0.031278 |
| 241 | Ribose 1-phosphate | 1.033051931 | 0.030753 |
| 242 | Antibiotic G-418 | 1.030576563 | 0.030321 |
| 243 | Inosine | 1.028546675 | 0.030713 |
| 244 | 13-L-Hydroperoxylinoleic acid | 1.026778686 | 0.037483 |
| 245 | Quinacrine | 1.025553602 | 0.03895 |
| 246 | S_-Abscisic acid | 1.024678938 | 0.032397 |
| 247 | Glycyl-leucine | 1.024173118 | 0.03246 |
| 248 | 6-Hydroxynicotinic acid | 1.022482504 | 0.031847 |
| 249 | Taurohyocholate | 1.020990751 | 0.032431 |
| 250 | Phenylbutazone | 1.020845271 | 0.03425 |
| 251 | trans-Cinnamate | 1.020047388 | 0.035451 |
| 252 | Linoleic acid | 1.016856376 | 0.038664 |
| 253 | Phenylethylamine | 1.015666117 | 0.033181 |
| 254 | dCDP | 1.007954831 | 0.035314 |
| 255 | Citramalic acid | 1.006453707 | 0.036762 |
| 256 | L-Methionine S-oxide | 1.001789082 | 0.038015 |
| 257 | Galactosylglycerol | 1.001683144 | 0.036404 |
| 258 | S-Methyl-L-methionine | 1.001184511 | 0.036222 |
| 259 | Styrene Oxide | 1.000858226 | 0.038606 |

Note: The differentially metabolites in hepatic tissue were flitered based on VIP>1.0 and P <0.05.
